# Consumption of Vegetables Is Associated with Systemic Inflammation in Older Adults

**DOI:** 10.3390/nu14091765

**Published:** 2022-04-23

**Authors:** Konstantinos-Georgios Papaioannou, Fawzi Kadi, Andreas Nilsson

**Affiliations:** School of Health Sciences, Örebro University, 70182 Örebro, Sweden; konstantinos.papaioannou@oru.se (K.-G.P.); fawzi.kadi@oru.se (F.K.)

**Keywords:** aging, dietary habits, inflammatory biomarkers, abdominal obesity, nutrition, sedentary time, physical activity

## Abstract

It is hypothesized that healthy diets rich in fruits and vegetables (FV) can modulate the inflammatory status in older adults. However, to determine the actual impact of FV on inflammatory status, adiposity level and objectively assessed physical activity (PA) behaviors need to be considered. The aim of the present study was to explore associations between FV intake and biomarkers of systemic inflammation in older adults. Based on a sample of 233 older adults (65–70 years old), the following inflammatory biomarkers were assessed: C-reactive protein (CRP), fibrinogen, interleukin-6 (IL-6), IL-10, IL-18, and monocyte chemoattractant protein-1 (MCP-1). FV intake was assessed by self-report, and PA behaviors encompassing time spent sedentary and in moderate-to-vigorous PA (MVPA) were determined using accelerometers. Associations between FV intake and inflammatory biomarkers were analyzed using stepwise linear regression models while adjusting for several covariates, including health-related food groups, adherence to the MVPA guidelines, total sedentary time, and waist circumference. While no significant associations were observed for the total FV intake, the vegetable intake was inversely associated with levels of IL6 (β = −0.15; *p* < 0.05). In contrast, fruit intake was not associated with any inflammatory biomarker. In conclusion, our findings indicate beneficial associations between vegetable intake and levels of a pro-inflammatory biomarker in older adults, which strengthens public health efforts to promote vegetable-rich diets in older adults to mitigate age-related systemic inflammation.

## 1. Introduction

Several reports have shown that diets characterized by low fruit and vegetable intake are associated with mortality [1], cardiovascular diseases [2], and type 2 diabetes mellitus [3,4]. Moreover, lowered cardiometabolic risk has been associated with higher consumption of either fruits [5], vegetables [6], or both fruits and vegetables (FV) [7,8]. Therefore, diets rich in FV are globally recommended by major health organizations in order to prevent the burden of chronic diseases and promote health [9].

During aging there is an elevated risk of cardiometabolic abnormalities, where a chronic inflammatory state characterized by slight elevations in the circulating levels of pro-inflammatory biomarkers has been implicated in the pathogenesis of age-related diseases [10]. In this respect, the potential of FV to mitigate age-related chronic systemic inflammation has received a particular attention [11,12]. Nonetheless, previous experimental work examining the effect of FV on established and novel biomarkers of systemic inflammation revealed conflicting results. For example, while a reduction in the level of the established inflammatory biomarker, C-reactive protein (CRP), was observed following increased FV intakes in one study [13], no corresponding effects were evident in other studies [14,15,16]. Further, although CRP levels were unaltered, changes in the level of some novel inflammatory biomarkers (e.g., TNF-related apoptosis-inducing ligand) [15] elucidate the complex interplay between diet and the inflammatory environment. Likewise, a compilation of observational studies [12] revealed conflicting results, where associations between FV intake and inflammatory biomarkers have been demonstrated in some [17,18,19] but not all studies [14,16,20].

Importantly, in order to determine the actual role of FV it is necessary to assess the potential confounding impacts of physical activity behaviors. Indeed, it has been shown that time spent in moderate-to-vigorous physical activity (MVPA) [21,22] may reduce levels of pro-inflammatory biomarkers, whereas time spent sedentarily has been detrimentally associated with systemic inflammation in older adults [23,24]. Additionally, whether any beneficial effect on systemic inflammation is driven by fruit or vegetable consumption needs clarification. Indeed, previous studies have shown beneficial effects on metabolic health outcomes driven by vegetables [6,25], fruits [4], or a combination of both [7,8]. Therefore, it can be hypothesized that variations in the nutritional composition of fruits and vegetables lead to different impacts on inflammatory biomarkers. Finally, although low FV intakes are commonly reported in aging populations [26], there are relatively few studies specifically targeting older adults when depicting the role of FV on systemic inflammation. Therefore, the aim of the present study was to explore the associations between intakes of fruits and vegetables and biomarkers of systemic inflammation in older adults, while considering objectively assessed PA behaviors.

## 2. Materials and Methods

### 2.1. Participants

Local advertisement was used to recruit the study sample. Data were collected from 252 community-dwelling older adults during year 2018. To be eligible for inclusion, participants had to be between 65 and 70 years old, free from overt diseases such as diabetes mellitus and coronary heart disease, with no mobility disabilities, and with no reported anti-inflammatory treatment. All participants provided written informed consent, and all research procedures were conducted in accordance with the principles set by the Declaration of Helsinki. The Swedish Ethical Review Authority approved the study (Dnr 2017/511).

### 2.2. Assessment of Dietary Intake

The total number of FV servings was determined based on the following questions from the Swedish national survey on health and living conditions [27]: how often do you eat fruit and berries, including all types of fruit and berries (fresh, frozen, preserved, juices, compote, etc.)? how often do you eat vegetables and root vegetables, including all types of vegetables, legumes, and root vegetables except potatoes (fresh, frozen, preserved, stewed, vegetable juices, vegetable soups, etc.)? Fixed answering categories ranged from less than one serving per day to up to 5 servings per day or more. In addition, a 90-item food-frequency questionnaire (FFQ) was used to determine total energy intake and intakes of health-related food groups, including whole-grains, fish, red or processed meats, fried potatoes, desserts and sweets, and sugar-sweetened beverages, as previously described [28].

### 2.3. Assessment of Anthropometry

Body height and weight were determined by standard procedures. Waist circumference (WC) was determined to the nearest 0.1 cm at the midpoint between the iliac crest and the lower costal margin using measuring tape. Participants were classified with abdominal obesity according to established criteria for metabolic risk (WC ≥80 cm for women and ≥94 cm for men) [29].

### 2.4. Assessment of Inflammatory Biomarkers

Blood samples were collected by venipuncture after an overnight fast. Fully automated immunoturbidimetric assay was used to determine high-sensitivity CRP. Fibrinogen was determined using an automated immunoassay method (Dako, Glostrup, Denmark). IL-6, IL-10, IL-18, and MCP-1 were analyzed using the Olink Proseek Multiplex Inflammation panel (Olink, Uppsala, Sweden), as previously described [23]. Briefly, antibodies coupled to oligonucleotides are bound to the target protein, a new PCR target sequence is formed by a proximity-dependent DNA polymerization event, and the resulting sequence is detected and quantified by RT-PCR.

### 2.5. Assessment of Physical Activity Behaviors

Participants wore an accelerometer (Actigraph GT3x, Pensacola, FL, USA) around the waist for a week, as previously described [23]. The average time (min) spent sedentary and in MVPA per day was retrieved based on established accelerometer count cut points [30]. Participants averaging 22 min of MVPA per day, which approximates 150 min per week, were classified as adhering to the MVPA guideline. Time spent sedentary was expressed in relation to total registered time per day.

### 2.6. Assessment of Other Covariates

Information about prescribed medication use, tobacco use (current, past, and never), and education level (university/college, high-school, and secondary school) were self-reported.

### 2.7. Statistical Analysis

Data are presented as means ± SD, unless otherwise stated. Data were examined for normality and transformed to fit a normal distribution when necessary. Differences in general characteristics were analyzed by Chi-square test. Multiple linear regression analysis was employed to assess the independent associations between intakes of fruits and vegetables alone and in combination and levels of each inflammatory biomarker. Due to different original units, the inflammatory biomarkers were expressed in standardized units prior to analysis. A stepwise backward elimination model was employed with a probability threshold for removal set to *p* > 0.1. Besides FV intake, the model included the following covariates: age, sex, education level, tobacco use, medication use, adherence to MVPA guideline, total sedentary time, waist circumference, total energy intake, use of dietary supplements, intakes of whole-grains, fish, red or processed meats, fried potatoes, desserts, sweets and sugar-sweetened beverages. Each variable fulfilling criteria for removal was eliminated in a stepwise manner. At the final step, only variables below removal threshold were retained. Assumptions behind regression analysis, including multicollinearity between independent variables, were checked. In the case of a significant association, differences in the levels of inflammatory biomarkers were further analyzed between groups of fruits and vegetables alone or in combination based on intake levels related to health benefits [1,31]. Based on an alpha level of 0.05, the current study sample allows for small to moderate effect sizes to be detected with a power of ≥80%. All statistical analyses were performed using SPSS version 27.0 (IBM Corp., Armonk, NY, USA).

## 3. Results

Information on diet, inflammation, and other covariates was collected for a total of 86 men (67 ± 1.5 yrs; 179 ± 7 cm; 81± 11 kg) and 147 women (67 ± 1.6 yrs; 164 ± 6 cm; and 64 ± 10 kg). Data on the proportions of participants with abdominal obesity, being physically active, use of tobacco and medication, and education level are shown in Table 1.

The assessment of FV consumption indicated an intake of 2.6 ± 1.8 and 3.4 ± 1.7 servings/day for men and women, respectively. Intakes for fruits and vegetables separately were 1.3 ± 1.1 and 1.2 ± 0.9 servings/day for men. Corresponding intakes in women were 1.8 ± 1.2 and 1.6 ± 0.9 servings/day for fruits and vegetables, respectively. Total energy intake was 2099 ± 586 kcal and 1542 ± 455 kcal in men and women, respectively. Fifty-four percent (men: 41%, women 62%) of the participants reported use of dietary supplements.

The levels of inflammatory biomarkers in men and women are shown in Table 2. The levels of IL-18 and IL-10 were significantly higher in men compared to women (*p* < 0.05).

A regression analysis with stepwise backward elimination revealed no significant relationships between levels of the examined inflammatory biomarkers and FV servings or fruit servings alone. In contrast, the number of vegetable servings was significantly associated with levels of IL-6 (β = −0.15, 95% CI: −0.30 to −0.01) in the final model retaining sex, waist circumference, and time spent sedentary. No relationships with other inflammatory biomarkers were observed.

**Figure 1:**
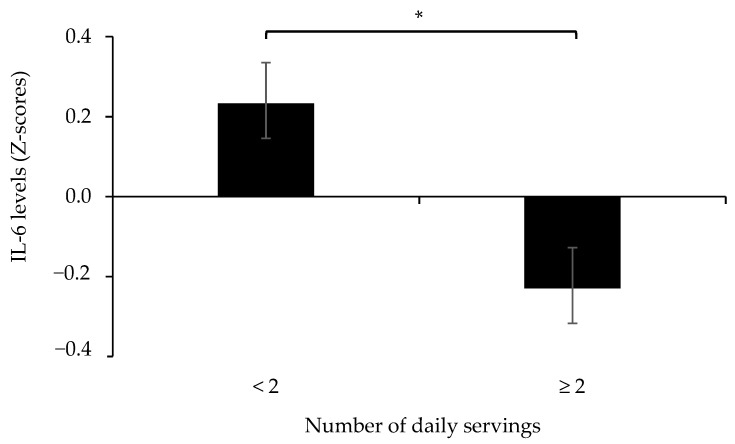
The levels of IL-6 (z-scores) in participants with lower (<2) and higher (≥2) numbers of daily vegetable servings. * *p* < 0.05.

Furthermore, we compared IL-6 levels between participants having a vegetable intake of ≥2 servings/day and those with lower intake. The analysis showed that participants in the lower intake group had higher (*p* < 0.05) IL-6 levels compared to those with a vegetable intake of ≥2 servings/day (Figure 1). No other differences in levels of inflammatory biomarkers were observed for groups of vegetable intake.

## 4. Discussion

In the present study on a population of older adults, a higher intake of vegetables but not fruits was associated with lower levels of the pro-inflammatory biomarker IL-6 regardless of the potential impacts of several health-related food groups as well as physical activity behaviors. The findings from our study also suggest the beneficial impact of vegetable consumption on biomarkers of systemic inflammation in older adults independently of adiposity level.

Few studies have reported on associations between inflammatory markers and specific food groups [12,32,33], with a particular scarcity of data in the elderly population. Previously, reports have shown inverse relationships between biomarkers of inflammation in older adults and intakes of FV [17,18,19] and vegetables alone [34]. Our data revealed a significant impact of vegetables but not fruits on the levels of the inflammatory biomarker Il-6, which is in line with data showing inverse relationships between vegetable intake and IL-6 levels in middle-aged [33] and older adults [34]. Our finding is further strengthened by the fact that adiposity level was accounted for in the present analysis. This is important as biomarkers of systemic inflammation are related to indicators of adiposity in older adults even after controlling for variation in physical fitness level [35]. As previous studies have highlighted the protective role of vegetables on the likelihood of having the metabolic syndrome [6] and type 2 diabetes [4,36], our findings are suggestive of an interplay between systemic inflammation and metabolic health, where adequate vegetable intake may promote a favorable meta-inflammatory environment.

Although the exact number of vegetables for optimal effects on the inflammatory environment is yet to be determined, our study revealed a lower IL-6 level in older adults with a vegetable intake of ≥2 servings/day compared to those with lower intakes. This indicates the benefits of vegetables on the inflammatory environment, adding to the body of evidence suggesting a vegetable intake of 2–3 servings per day for general health benefits [1,31]. The exact mechanisms underlying the protective effects of vegetables on inflammatory and metabolic health status are still unknown. However, their content in antioxidants, fiber, vitamins, phytochemicals, and the low glycemic index of vegetables likely act in synergy to impact on several biological factors related to the inflammatory environment [37]. Notably, the present study shows that fruit intake was not associated with inflammatory biomarkers. In this respect, it has been hypothesized that compared to vegetables, the relatively higher amount of fructose in fruits may mitigate their protective health effects [38]. Further studies are warranted in order to clarify the general role of different types of fruits and the proposed impact of their varying nutritional composition, including their fructose content. In addition, the dose response relationship between FV and systemic inflammation may vary depending upon the intake range of the study sample and the selected inflammatory biomarkers. For example, a beneficial impact of FV on CRP could be demonstrated only after reaching 8 servings per day [13], which indicates that modulation of some inflammatory biomarkers may require FV intakes that are substantially higher than commonly reported in adult populations. Indeed, the FV intake in our study sample reflects what has been reported for Swedish older adults, where only a minority reaches the recommended five servings per day [39]. Therefore, the ability to capture the full immunomodulatory potential of FV would likely require study samples with wide intake ranges. Finally, in order to capture the complexity of the inflammatory systemic environment in older adults and its links to dietary habits, further investigations, including a comprehensive set of inflammatory biomarkers, are warranted.

The robustness of our findings relies on the inclusion of objectively assessed PA behaviors when modelling the associations between FV intake and systemic inflammation. Indeed, the influence of time spent in MVPA and in sedentary behaviors on the regulation of the inflammatory environment in older adults has previously been evidenced [21,22,23]. Therefore, our study emphasizes the importance of considering the separate impacts of both diet and PA behaviors in order to mitigate the age-related elevations of circulating inflammatory biomarkers. Furthermore, a number of other food groups commonly used to characterize healthy dietary patterns [40] were considered in our analysis, which further supports the health-enhancing potential of a plant-rich diet.

Nevertheless, the following considerations should be taken into account when interpreting our data: (a) causality cannot be determined as it is based on a cross-sectional analysis; (b) due to characteristics of the study sample, caution should be taken when generalizing the findings to broader populations of older adults; and (c) although the potential influence of several covariates was considered, residual confounding may still be present.

## 5. Conclusions

In conclusion, the present study demonstrates that a higher intake of vegetables but not fruits is associated with lower levels of the pro-inflammatory biomarker IL-6 regardless of the physical activity behaviors in older adults, which strengthens public health efforts to promote vegetable-rich diets in older adults to mitigate age-related systemic inflammation.

## Figures and Tables

**Table 1:** The general characteristics of the study population.

	Men (*n* = 86)	Women (*n* = 147)
Abdominal obesity (%)	50	50
Medication (%)	42	48
Physically active (%)	80	84
Tobacco use (%)		
Never	43	54 *
Former	47	43
Current	10	3
Education level (%)		
University/College	52	71 *
High school	34	22
Secondary school	14	7

* *p* < 0.05.

**Table 2:** The biomarkers of inflammation in men and women.

Inflammatory Biomarkers	Men (*n* = 86)	Women (*n* = 147)
CRP (mg/L) ^a^	1.09 ± 1.97	1.07 ± 2.14
Fibrinogen (mg/L)	3.17 ± 0.54	3.17 ± 0.57
IL-6 (au)	3.40 ± 0.58	3.28 ± 0.59
IL-18 (au)	8.25 ± 0.47	7.93 ± 0.53 *
IL-10 (au)	3.88 ± 0.42	3.70 ± 0.38 *
MCP-1 (au)	12.54 ± 0.42	12.45 ± 0.40

au: arbitrary units; ^a^ geometric mean; * *p* < 0.05.

## Data Availability

Data supporting reported results are available upon reasonable request and in accordance with the ethical principles.
